# Biomechanical control of lymphatic vessel physiology and functions

**DOI:** 10.1038/s41423-023-01042-9

**Published:** 2023-06-02

**Authors:** Veronique Angeli, Hwee Ying Lim

**Affiliations:** 1 Immunology Translational Research Programme, Yong Loo Lin School of Medicine, Department of Microbiology and Immunology, National University of Singapore, Singapore, Singapore; 2 Immunology Programme, Life Sciences Institute, National University of Singapore, Singapore, Singapore

**Keywords:** lymphatic vessel, biomechanical force, mechanosensing, mechanotransduction, human diseases, Immunology, Lymphatic vessels

## Abstract

The ever-growing research on lymphatic biology has clearly identified lymphatic vessels as key players that maintain human health through their functional roles in tissue fluid homeostasis, immunosurveillance, lipid metabolism and inflammation. It is therefore not surprising that the list of human diseases associated with lymphatic malfunctions has grown larger, including issues beyond lymphedema, a pathology traditionally associated with lymphatic drainage insufficiency. Thus, the discovery of factors and pathways that can promote optimal lymphatic functions may offer new therapeutic options. Accumulating evidence indicates that aside from biochemical factors, biomechanical signals also regulate lymphatic vessel expansion and functions postnatally. Here, we review how mechanical forces induced by fluid shear stress affect the behavior and functions of lymphatic vessels and the mechanisms lymphatic vessels employ to sense and transduce these mechanical cues into biological signals.

## Introduction

The advances in molecular, cellular, genetic, and imaging approaches in the past decade have led to the identification of specific lymphatic endothelial cell (LEC) markers, allowing the distinction between blood and lymphatic vessels and identification of the mechanisms controlling the development and stabilization of lymphatic vessels and their functions. Together, these discoveries have changed our perception of the lymphatic vasculature; namely, it is no longer believed to be a secondary vascular system but rather a system that is vital for human well-being and health. Indeed, the lymphatic system, with its extensive plexus of vessels and interconnected lymph nodes, plays essential roles in tissue homeostasis by clearing waste, in immunosurveillance by controlling immune cell trafficking and responses, and in organ morphogenesis by coordinating tissue regeneration [1, 2]. Moreover, the importance of the functional roles of lymphatic vessels in humans diseases is being increasingly recognized. Until recently, the traditional pathology associated with malfunctions in lymphatics was lymphedema. However, with increasing research on lymphatics, novel functional roles of the lymphatic vasculature in diseases have been discovered. As such, the number of human diseases or disorders associated with lymphatic defects has increased, and inflammatory bowel diseases, eye diseases, neurological disorders, obesity, and cardiovascular diseases are now included [2]. These new findings imply that restoring optimal lymphatic function may be a promising nonconventional strategy to managing these diseases. Thus, a deeper understanding of the mechanisms regulating lymphatic physiology and functions will be useful for the design of such an approach. In adult tissues, lymphatic vessel phenotype and behavior has been shown to be shaped by biochemical signals such as vascular endothelial growth factors (VEGFs). However, other cell-extrinsic factors likely impact lymphatic vessels. For example, cells commonly experience different mechanical forces triggered by changes in the magnitude or type of fluid shear stress, extracellular matrix composition, and stiffness. Likewise, emerging evidence indicates that lymphatics can also sense and transduce nearby mechanical forces into signals that regulate molecular pathway gene expression, controlling LEC cytoskeleton remodeling, proliferation, migration, the cell cycle, permeability and other functions [3–5]. The effect of changes in fluid osmolarity, hydraulic pressure, temperature, matrix composition and stiffness in the microenvironment has been reviewed [6, 7]. Therefore, in this review, we focus our attention on mechanical forces generated by interstitial fluid flow experienced by lymphatic vessels and describe the responses induced by this type of mechanical force in lymphatics, as well as the underlying sensing and mechanotransduction mechanisms, after summarizing the biological functions of lymphatic vessels in health and diseases.

## Biological functions of lymphatic vessels and implications in diseases

Here, we provide a brief overview of conventional and newly discovered biological functions of lymphatic vessels and their implications in health and diseases, as this topic has been well described in recent reviews [1, 2].

### Lymphatic fluid and lipid transport

**Fig. 1:**
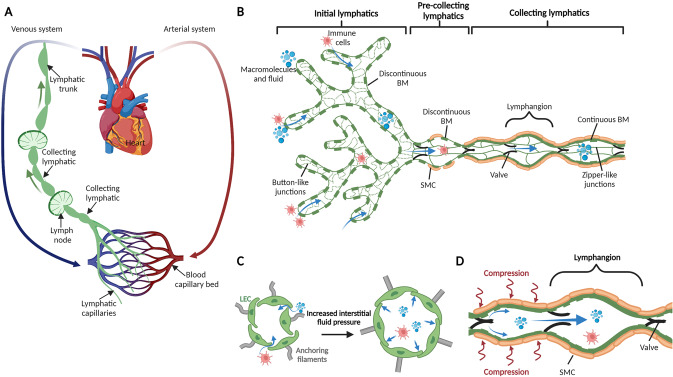
Structure and function of the lymphatic system. **A** The lymphatic vasculature (green) forms part of the circulatory system. Fluid that extravasates from the blood capillary bed into the tissue interstitium is absorbed into initial lymphatics vessels and flows through larger collecting lymphatic vessels that actively transport lymph fluid into draining lymph nodes before returning into the venous system via the thoracic duct. **B** Interstitial fluid, macromolecules and immune cells leave the tissue interstitium to enter discontinuous button-like initial lymphatic vessels that lack a continuous basement membrane. Collecting lymphatic vessels have a continuous basement membrane, smooth muscle cell coverage to provide contractile activity to assist blood flow and intraluminal valves to prevent lymph backflow. Collecting LECs are organized into tight continuous zipper-like junctions and do not absorb fluid from surrounding tissues. **C** Initial lymphatic vessels are composed of overlapping LECs that allow interstitial components to enter the vessels when interstitial pressure is high. The overlapping cells also act as valves, preventing fluid from leaking out. Anchoring filaments connect LECs to the surrounding extracellular matrix and facilitate fluid, macromolecule and cell entry into initial lymphatic vessels. **D** The collecting lymphatic vessels are composed of several lymphangions that propagate lymph flow. Coordinated contraction/expansion of each lymphangion and opening/closing of intraluminal valves ensure efficient lymph transport. LEC lymphatic endothelial cell, BM basement membrane, SMC smooth muscle cell. Created with BioRender.com

Lymphatic vessels have long been recognized to function in the transport of interstitial fluid from the tissue to the draining lymph node (LN) and back to the venous system via the thoracic duct (Fig. 1). Thus, defective lymphatic drainage leads to fluid accumulation and subsequent tissue swelling. This process results in a disorder known as lymphedema, and it can be either congenital (primary lymphedema) or acquired (secondary lymphedema) [8]. Numerous genes associated with primary lymphedema have been identified, and they often overlap with genes implicated in the development of lymphatic vessels [2]. Secondary lymphedema occurs because of surgery, radiation therapy, infection or trauma that damages lymphatic vessels and compromises their draining function. Lymphedema is a progressive and chronic disease that lacks a cure [9, 10]. In addition to fluid transport, lymphatic vessels are also involved in lipid transport. Lacteals together with submucosal lymphatic vessels form the intestinal lymphatic vasculature and control the absorption of dietary fat. Briefly, dietary lipids are absorbed by enterocytes and transformed into large triglyceride-loaded lipoprotein particles, called chylomicrons. These lipoproteins are taken up by lacteals, blunt-ended initial lymphatics that transport them into the intestinal submucosal lymphatics and mesenteric collecting vessels. Intestinal then lymph drain to the mesenteric LNs and thoracic duct and further to the blood circulation [1]. Lacteals are surrounded by longitudinal villus SMCs, which facilitate efficient drainage of absorbed lipids by actively contracting and squeezing lacteals [11]. Because lacteals control dietary lipid absorption, they are also involved in regulating body weight [12–15]. Moreover, maintaining a healthy and functional lymphatic vasculature might be important for preventing metabolic diseases and obesity since a growing body of evidence indicates that lipid escape from leaky lymphatics can lead to fat accumulation [16–19]. Similarly, lymphatic vessels are involved in the transport of lipoproteins from the interstitial tissue to the blood [20–22]. The idea that lymphatic vessels function in the transport of lipoproteins from interstitial tissue to blood was proposed in the late 1980s [23], and this lymphatic function was revisited more recently by two separate groups. The study of Martel et al. [24] in *Chy* mice, a model of dermal lymphatic insufficiency, and our study [25] using wild-type mice in which lymphatic vessels were surgically excised provided the first clear, direct evidence that impaired lymphatic drainage significantly decreases the efficiency of cholesterol transport mediated by high-density lipoprotein (HDL) from the interstitium back to the blood circulation, a process known as reverse cholesterol transport (RCT). Excess cellular cholesterol can be highly toxic and eventually leads to cell death. RCT regulates the abundance of the intracellular cholesterol pool by removing excess cholesterol from peripheral tissues and transporting it to the liver via plasma for excretion [26]. We also further proposed that the transport of effluxed cholesterol from peripheral tissue to the circulation via lymphatics not only is passive as believed but also can be an active process dependent on HDL and scavenger receptor class BI (SR-BI) [25]. These findings support the concept that lymphatic vessels tightly regulate the clearance of cholesterol from the peripheral tissues through the transport of HDL-cholesterol [21]. Notably, this concept may also be true in humans based on a few case reports describing skin cholesterol deposition in primary lymphedema patients [27–31]. Because they can prevent cholesterol and other lipids from accumulating in tissues, lymphatic functions may play a role in cardiac- and metabolic diseases associated with lipid alterations, such as atherosclerosis and diabetes. Thus, strategies promoting efficient lymphatic drainage might be useful for these diseases [32–36].

### Lymphatic control of immune cell trafficking

In addition to fluid and lipid transport, lymphatic vessels transport soluble antigens and immune cells from peripheral tissues into regional LNs via afferent lymph (Fig. 1B). Classic cannulation studies carried out in humans and small and large animals have revealed that the major migrating cells in normal afferent lymph are T lymphocytes (80–90%), followed by antigen-presenting dendritic cells (DCs) and very small numbers of B lymphocytes, which together account for most of the remaining 10–15% of cells. The number of CD4-expressing T lymphocytes is much higher than that of CD8^+^ T cells [37–39]. Only low numbers of naive T cells are usually present in afferent lymph, and most of the T cells are antigen- experienced memory cells [40, 41], although a significant proportion of CD4^+^ T cells are T regulatory cells, as recently demonstrated using photoconvertible Kaede mice [42]. Advances in in vivo and in vitro methods to assess cell migration, such as time-lapse imaging in tissue explants and intravital microscopy [43, 44], have greatly contributed to elucidating the cellular and molecular mechanisms controlling lymphatic migration of DCs and T cells (as reviewed [43, 45, 46]). The chemokine receptor CCR7 expressed on the surface of DCs and its ligand CCL21 produced by lymphatic endothelial cells (LECs) [47–49] are the main molecules involved in DC migration via afferent lymphatics under steady-state and inflammatory conditions; CCR7 and CCL21 guide DCs through the interstitium toward afferent lymphatics and within the initial lymphatics toward collecting vessels [50–52]. Although CCR7 deficiency or blockade of CCL21 significantly impairs DC migration to draining LNs, other chemokines have been found to be involved in this process [43, 53, 54], including CXCL12 and CX3CL1, which bind to DC-expressed CXCR4 and CX3CR1, respectively [53, 54]. In contrast to leukocyte extravasation from blood vessels, which is a highly integrin-dependent process, DC migration into and through afferent lymphatics in steady state conditions is independent of DC-expressed integrins [55] and only becomes integrin-dependent when LECs upregulate ICAM-1 and VCAM-1 in response to inflammation [56, 57]. Similar to DCs, the CCR7-CCL21 pathway mediates T-cell migration from peripheral tissue via afferent lymphatics in steady state and in inflammation [58].

Lymphatic vessels in the LNs also play an important role in the trafficking and positioning of immune cells within the LNs, which are critical for proper immune responses [59]. In the LN, fibroblastic reticular cells are the major source of CCL21, and LECs lining the lymphatic sinus “ceiling” express high levels of a decoy receptor for CCL21, called CCRL1. This organization creates a CCL21 gradient supporting the migration of DCs into the LN medulla [60]. Subcapsular sinus LSCs have also been shown to control the entry of lymphocytes and soluble antigens into the LN parenchyma through the expression of plasmalemma vesicle-associated protein (PLVAP), which is normally restricted to fenestrated blood endothelial cells [61]. Finally, LN LECs control the egress of activated T cells such as effector T cells from LNs by releasing the signaling phospholipid sphingosine-1-phosphate (S1P) into the efferent lymph, which attracts T cells through its binding to the S1P receptor S1P1 [62, 63]. Notably, S1P is also produced by LECs in peripheral tissues and affects DC migration into draining LNs [63–65].

### Lymphatic regulation of T-cell responses

In addition to contributing to immunity through the transport of soluble antigens and migration of immune cells, lymphatic vessels have emerged as key regulators of T cells responses in the past decade (as reviewed [66]). Several studies in mice have reported that steady-state LN LECs contribute to peripheral T-cell tolerance by presenting endogenously expressed tissue-restricted antigens [67, 68] through MHC class I (MHCI) molecules and eliminating autoreactive CD8^+^ T cells [69–71]. LN LECs can also control the elimination of antigen-specific CD8^+^ T cells by cross-presenting exogenous antigens in MHCI molecules, which in turn leads to T-cell apoptosis [72]. Although several studies indicate that LN LECs can also impact peripheral CD4^+^ T-cell responses, further studies in different immunological settings are necessary to confirm this role [66]. Indeed, the study by Rouhani et al. [73] showed that LECs were incapable of presenting antigenic peptides via MHC class II (MHCII) since they do not express H2-M at steady state. In contrast, Dubrot et al. reported that in addition to inducing antigen-specific CD4^+^ T-cell tolerance by presenting peptide-MHCII complexes acquired from DCs, LECs endogenously express MHCII molecules through the IFN-γ-inducible promoter IV (pIV) of the MHC class II (MHCII) transactivator CIITA [74]. Recently, the same group provided evidence that the absence of MHCII expression in LN LECs in aging mice impairs peripheral CD4^+^ T-cell tolerance by promoting defective regulatory T cells and increasing effector T cells, which results in peripheral organ T-cell infiltration and autoantibody production [75], further supporting the potential role of LECs in peripheral CD4^+^ T-cell responses.

Furthermore, LECs have been shown to influence naive T-cell survival through the release of S1P, which stimulates mitochondrial function [76]. LN LECs are also capable of dampening T-cell activation and proliferation during inflammation either directly through the production of nitric oxide induced in response to T-cell cytokines [77] or indirectly by controlling the maturation of DCs [78, 79]. Thus, lymphatic vessels participate in important processes in immunosurveillance and immunomodulation by regulating immune cells, soluble antigen transport and T-cell responses. Lymphatic vessels have been shown to play active roles in infectious and inflammatory diseases, tissue transplants and cancer, which are often associated with the expansion of lymphatic vessels, through these immune functions [2].

In cancer, lymphatic vessels have been found to play a role in the spreading of solid tumor cells to distant organs by serving as channels connecting the primary tumors to LNs. During cancer progression, tumor cells and cells from the tumor environment produce growth factors that promote lymphatic cell proliferation, sprouting and enlargement in and around solid tumors, a process known as lymphangiogenesis. Tumor lymphangiogenesis correlates with metastasis and poor patient prognosis in several cancer types (as discussed [80]). For example, in melanoma patients, intratumoral lymphatics are associated with distant metastasis [81] and poor disease- free survival [82], and lymphatic vessel area is associated with poor overall survival [83]. Similarly, in colorectal, breast and lung cancers high lymphatic vessel density is an indicator of lymph node metastasis and associated with reduced overall survival [84–89]. In mice, lymphangiogenesis has also been shown to promote the dissemination of metastases to distal organs [90, 91]. Therefore, therapies aimed at blocking tumor lymphangiogenesis are currently being developed as promising approaches for the treatment of malignancies such as melanoma and colorectal cancer [92]. However, LECs embedded in the tumor environment may also modulate tumor immunity [93] and influence anticancer therapy responses. Notably, recent studies suggest that tumor-associated lymphatic vessels may exhibit positive and/or negative effects on tumor immunity depending on the tumor stage and the immunological environments of the tumor (which differ in cases of immune evasion/immune subversion or with immunotherapy) [66]. Tumor- associated lymphatic vessels are necessary for the initiation of antitumoral responses because they support DC trafficking [94, 95], and tumor-associated lymphangiogenesis in melanoma can potentiate immunotherapy by promoting T-cell infiltration [96]. However, LECs in the tumor environment can exhibit immunosuppressive functions that will conversely reduce antitumor immunity. During tumor progression, LECs have been shown to upregulate PD-L1 expression in response to IFNy secreted by tumor-specific CD8^+^ T cells and subsequently suppress T-cell accumulation in tumors [97, 98]. Although further investigations are needed to decipher the specific contributions of lymphatics to antitumor immunity, these findings reveal the potential utility of targeting lymphatic functions during tumor development. Moreover, it is unlikely that the regulatory roles of lymphatics will be limited to T cells given that LECs can potentially interact with a variety of immune cells depending on the tissue/organ in which they reside and shape their responses through the expression of multiple growth factors, cytokines and chemokines.

The finding that LECs in LNs affect medullary macrophages through production of the survival factor colony-stimulating factor-1 supports this idea [99]. Thus, the list of immune cells regulated by LECs is expected to grow. Furthermore, given that lymphatic vessels orchestrate immune responses and disposal of tissue fluid, proteins, and lipids, it is reasonable to postulate that lymphatic functions are important in many diseases associated with the accumulation of such biological waste and immune dysfunction, including cardiovascular diseases (e.g., myocardial infarction, atherosclerosis), ocular diseases (e.g., glaucoma), Crohn’s disease and neurological diseases (Alzheimer’s disease, Parkinson’s disease, stroke) [1, 100–102].

### Adult lymphatic vessel anatomy and plasticity

The lymphatic system is composed of an extensive network of vessels and interconnected lymph nodes. This unidirectional vascular network includes blind-ended capillaries or initial lymphatic vessels and larger lymphatic vessels, known as the collecting lymphatic vessels, on which the initial lymphatics converge (Fig. 1). Both lymphatic types are lined with LECs that express platelet endothelial cell adhesion molecule (PECAM-1 or CD31), a marker shared with blood endothelial cells (BECs), the homeobox transcription factor prospero-related homeobox 1 (PROX1) and the receptor tyrosine kinase vascular endothelial growth factor (VEGF) receptor-3 (VEGFR-3) [103, 104]. Initial lymphatic vessels can be identified by the expression of podoplanin and lymphatic vessel hyaluronan receptor 1 (LYVE-1) [105] and the absence of mural cells (e.g. pericyte or smooth muscle cells). They are characterized by a discontinuous basal membrane and specialized button-like cell junctions that allow interstitial fluid, lipids, soluble antigens and immune cells to have easy access to the lumen of the initial lymphatics [106] (Fig. 1B). Moreover, fluid enters through the opening of overlapping flaps localized between the button junctions of adjacent LECs that are operated by anchoring filaments that tether the initial lymphatics to surrounding extracellular matrix and sense changes in interstitial pressure [107, 108] (Fig. 1C). Under conditions of increased interstitial pressure, the anchoring filaments pull the LECs and open the overlapping flaps, which allows the uptake of lymph tissue fluid [109, 110]. Once the vessel is filled, the increasing luminal pressure closes the overlaps, thus preventing leakage of lymph out of the vessels [111]. The overlapping flaps also serve as lymphatic valves to prevent backflow of fluid from the lymphatic lumen (Fig. 1C). To our knowledge, few molecules regulating the connections of LECs with the anchoring filaments and extracellular matrix have been identified thus far. Among those that have been identified, the elastic microfibril-associated protein Emilin1 is highly expressed in LECs and is a component of the anchoring filaments in lymphatic vessels [112]. Deficiency in murine Emilin1 leads to structural defects in lymphatic vasculature, including reduction in anchoring filaments, lymphatic hyperplasia and disorganization, which impair lymphatic drainage and lead to lymph leakage. Collecting vessels equipped with zipper-like junctions also express podoplanin but downregulate LYVE-1 expression. They are covered with a basement membrane and circumferential smooth muscle cells and have luminal valves that propel and maintain unidirectional lymph flow to draining lymph nodes and eventually into the venous circulation via the thoracic duct (Fig. 1B, D) [113, 114]. Unlike the blood system, the lymphatic system does not possess a central pump; instead, lymph is propelled against an overall hydraulic pressure gradient from interstitial spaces to central veins thanks to two pumping mechanisms, which rely on extrinsic forces (contraction of surrounding skeletal muscles and arterial pulsations) or the intrinsic rhythmic contractility of lymphatic muscle cells. Coordinated contraction of muscle cells facilitates transport of lymph back to the blood circulation [115]. Notably, lymphatic contraction malfunctions, barrier dysfunction and valve defects in collecting vessels have been associated with pathologies either directly involving the lymphatic system, such as primary and secondary lymphedema, or indirectly involving the lymphatic system, such as inflammation, obesity, metabolic syndrome, and inflammatory bowel disease [116].

Recent progress in lymphatic biology research has unveiled the plasticity of adult lymphatic vessels, which can actively sense and adapt to changes within their tissue environment [117]. Notably, inflammation triggered by pathogen and nonpathogen (damaged cells, irritants) stimuli has been shown to alter the structure and function of lymphatic vessels in adult mammals [118]. Postnatal lymphangiogenesis, the formation of new lymphatic vessels from pre-existing ones, has been shown to occur in experimental and clinical inflammatory conditions including renal [119, 120] and corneal transplant rejection [121–123], inflammatory bowel disease [124–127], rheumatoid arthritis [128–130], chronic airway inflammation [131, 132], atopic dermatitis and psoriasis [133–135]. To date, several factors have been reported to promote lymphatic vessel expansion in vitro and in animal models including VEGF-A, VEGFC, VEGF-D, FGF-2, PDGF, IGF-1, IGF-2, angiopoietin-1, and HGF [107, 136], and the list continues to rapidly expand. VEGF-C and its receptor VEGFR-3 are the main drivers of both developmental and inflammatory lymphangiogenesis (as reported [117]) and are believed to be the most promising therapeutic targets for the treatment of human lymphedema [136]. In addition, cytokines and chemokines can also regulate lymphatic expansion directly or indirectly by inducing VEGF-C [137, 138].

Despite the undisputable role of the above biochemical signals in controlling lymphatic expansion and behavior during physiological and pathophysiological conditions, it is conceivable that lymphatic vessels also respond to mechanical signals produced by changes in microenvironment surrounding lymphatic vessels, which in turn impacts lymphatic vasculature and functions.

### Biomechanical control of lymphatic physiology and functions

Lymphatics are constantly exposed to shear stress produced by fluid flow at their luminal and abluminal surface. During lymph fluid uptake by the initial lymphatics, LECs may encounter interstitial fluid flow with in basal-to-luminal (or transmural) direction at their intercellular junctions. The lymph fluid then flows into the downstream collecting lymphatics, inflicting laminar shear stress on the luminal surface of capillary LECs. The collecting lymphatics transport lymph to the draining lymph node and back to the venous circulation. LECs lining the collecting vessels may more frequently face oscillatory flows. Moreover, lymphatic vessels are surrounded by a wide range of supportive structural cells, such as SMCs, in collecting vessels and diverse extracellular matrix components forming the basement membrane of the vessels and filling the interstitial space, which can exert other physical forces, such as stretching and stiffness forces. Thus, it is proposed that lymphatic vessels are exposed to several physical forces, and they are capable of translating this mechanical information into biological responses such as vessel remodeling, expansion, and stabilization, which are important for maintaining their physiological functions [4–6, 139]. Alterations in mechanical forces are often observed during disease and may result in abnormal lymphatic structure and function and thus contribute to pathology.

**Fig. 2:**
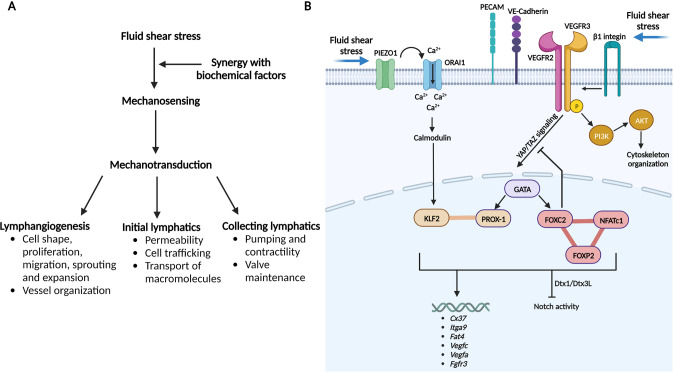
Fluid shear stress induced changes in lymphatics. **A** Effects of fluid shear stress on lymphatic vasculature and function. **B** Mechanosensory and mechanotransduction pathways in lymphatic endothelial cells. Lymphatic endothelial cells sense shear stress produced by interstitial fluid flow via 2 possible mechanisms. The mechanosensory complex, including PECAM, VE-cadherin, VEGFR2 and VEGFR3, senses fluid shear stress and activates the PI3K/Akt pathway, induces cytoskeleton reorganization and regulates YAP/TAZ signaling. In addition to effects on this complex, flow sensing leads to the activation of VEGFR3 phosphorylation via its ligand VEGF-C or interaction with β1 integrin and induces the downstream pathway activation mentioned above and the regulation of key genes, including the master transcription factors PROX-1, FOXC2, GATA2 and others depicted in this schematic diagram. The second mechanosensor in LECs, PIEZO1, activates the membrane-bound calcium channel ORAI1, leading to intracellular calcium entry. The major intracellular calcium sensor calmodulin forms a protein complex with KLF2, which subsequently drives lymphatics-related downstream gene upregulation or downregulation. Abbreviations: PIEZO1 Piezo type mechanosensitive ion channel component 1, ORAI1 calcium release-activated calcium channel protein 1, PECAM platelet and endothelial cell adhesion molecule, VE-cadherin vascular endothelial cadherin, VEGFR2 vascular endothelial growth factor receptor 2, VEGFR3 vascular endothelial growth factor receptor 2, YAP/TAZ yes-associated protein (YAP) and transcriptional coactivator with PDZ-binding motif (TAZ), PI3K phosphoinositide 3-kinases, AKT serine/threonine-protein kinase, GATA2 GATA binding protein 2, KLF2 Krüppel-like factor 2, PROX-1 prospero homeobox protein 1, FOXC2 Forkhead box protein C2, NFATc1 calcineurin/NFAT, FOXP2 Forkhead box protein P2, Cx37 connexin37, Itga9 integrin alpha- 9/beta-1, Fat4 FAT tumor suppressor homolog 4, Vegfc vascular endothelial growth factor C, Vegfa vascular endothelial growth factor A, Fgfr3 fibroblast growth factor receptor 3, Dtx1 deltex E3 ubiquitin ligase 1, Dtx3 L deltex E3 ubiquitin ligase 3L. A letter P in the yellow circles indicates phosphorylation. Created with BioRender.com

Therefore, it is critical to understand which aspects of lymphatic vessel biology are influenced by mechanical forces and to characterize the mechanisms by which lymphatics sense and respond to physical forces. In this section of the review, we first focus our attention on describing in vitro and in vivo studies supporting the responses to fluid-induced mechanical forces in lymphatics followed by a description of mechanotransduction and sensing mechanisms employed by lymphatics in response to fluid shear stress (FSS) (Fig. 2).

### Responses to flow-induced mechanical forces in lymphatics

LECs are subjected to fluid shear stress (FSS). Unlike blood vessels, which are controlled by a central pump and subjected to high FSS, initial lymphatics are exposed to interstitial fluid flow and thus lower FSS, approximately 10-fold less than blood vessels [140]. The hydrostatic and osmotic pressure between lymphatic vessels, blood vessels and the interstitial space guide the uptake of interstitial fluid and resulting forces (known as Starling forces) [141, 142]. Because collecting lymphatic vessels have an intrinsic pumping capacity and smooth muscle cell coverage (Fig. 1A), they experience greater FSS than the initial lymphatics, which ensures efficient transport of the lymph back to the venous circulation [116].

In vivo, LECs are exposed to a broad range of shear stresses depending on the tissue location of the lymphatic vessels and the tissue conditions (normal versus lymphedema). For example, LECs experience shear stress ranging from 0 to 12 dynes/cm^2^ in rat mesenteric prenodal lymphatics [140] and up to 40 dynes/cm2 in models of lymphedema [143]. Shear stress can be steady laminar (where fluid moves in one direction at a steady magnitude), disturbed laminar (where flow separates, recirculates and subsequently reattaches), oscillatory (where laminar flow fluid moves in a bidirectional manner) or turbulent (where flow is chaotic and moves in all directions) [5]. Both oscillatory and turbulent flows are often classified as disturbed.

Early studies in the 2000s unveiled that lymphatic expansion and remodeling can be triggered by FSS, which acts as a critical lymphangiogenic mediator by controlling LEC migration, VEGF- C expression, and initial lymphatic formation (Fig. 2A) [144]. An in vitro study using 3D collagen gel cultures showed that interstitial flow is a morphogenetic mediator of LEC organization and stabilization [145] (Fig. 2A). More recent studies have confirmed and further extended the effects of FSS on LECs to include effects on LEC shape, alignment, migration, and cell cycle and, importantly, revealed that these effects depend on the direction, magnitude, velocity and strength of pump pulses [146]. For example, under 4 dynes/cm^2^ laminar shear stress (LSS), LECs elongate and form stress fibers aligned with the flow direction, whereas LECs adopt a more cuboidal cell shape under 4 dynes/cm2 ¼ Hz oscillatory shear stress (OSS), and the amount of short perinuclear F- actin stress fibers increases; furthermore, their alignment is less dependent on flow direction [147]. Notably, the formation of cuboidal cells upon exposure of LECs to OSS resembles that of lymphatic valve cells in vivo, whereas the elongated and aligned LECs that form under LSS tend to show lymphangion cell morphology [147]. In an in vivo model of loss or gain of interstitial flow, Planas- paz et al. [139] showed that increased fluid can affect LEC stretching and proliferation (Fig. 2A).

Interestingly, the dynamic changes in LEC morphology observed in vitro in response to LSS have also been reported to alter lymphatic barrier function through a mechanism dependent upon Rac1-mediated actin dynamics [148]. In line with the effect of FSS on lymphatic physiological responses, sensitivity of LECs to shear stress has been postulated to be essential for adapting lymphatic function to meet tissue drainage needs and thus maintain body fluid homeostasis. Baeyens et al. [149] proposed the concept of the FSS set point, which assumes the existence of a preferred range of shear stress that determines vascular remodeling.

Consistent with the low and high levels of shear stress experienced by lymphatic and blood vasculature, respectively, the set point of BECs ranged between 10 and 20 dyne/cm^2^, as determined in vitro based on cell alignment and NF-kB translocation, which is a well-studied response associated with vessel stabilization and suppression of inflammatory pathways [150]. On In contrast, LECs are markedly more sensitive, with a set point of 4–10 dyne/cm^2^, which is in the range of shear stress values observed in the rat mesenteric lymphatic collecting vessels [140]. The high sensitivity of LECs to low levels of shear stress is determined by the increased expression of VEGFR3 in LECs compared to BECs. Reducing VEGFR3 expression in LECs increases the FSS setpoint, whereas increasing VEGFR3 expression in BEC decreases the FSS setpoint [149].

Further supporting the involvement of flow-induced mechanical forces in maintaining the physiological functions of lymphatics, a study using in vitro and in vivo models revealed that transmural flow enhances lymphatic permeability, transport of macromolecules and DC migration into lymphatics [151] (Fig. 2A). This DC migration was found to be accompanied by increased lymphatic expression of the chemokine CCL21, which is essential for the homing and docking of CCR7 expressing DCs to initial lymphatics as well as increased expression of adhesion molecules such intercellular adhesion molecule-1 and E-selectin. Moreover, the authors used an experimental model of lymphedema in which lymphatic drainage is markedly decreased, and in the model, lymphatic endothelial expression of CCL21 was nearly absent. This study suggests that FSS can act as an early signal for initial lymphatics to regulate immune cell trafficking and soluble antigen transport from the interstitial tissue to the draining lymph nodes. This regulatory mechanism may thus impact immunity and the progression of inflammatory diseases.

The effects of FFS on lymphatic function are not limited to initial lymphatics and also apply to collecting lymphatic vessels (Fig. 2A). The contractility of collecting vessel SMCs contributes to the pumping function of the vessels and can rapidly adapt to changes in the microenvironment resulting, for example, from flow-induced wall shear stress. Low nitric oxide (NO) concentrations produced by endothelial NO synthase (NOS), such as those associated with pulsatile flow from spontaneous contractions, have been shown to increase contraction amplitude [152] whereas higher NO concentrations produced through the activation of inducible NOS and inflammatory cytokines [153, 154] inhibit both contraction frequency and amplitude [116]. Interestingly, a study revealed FSS as a modulator of NO release and lymphatic pump function in contractile lymphatics [155]. A more recent study by Kornuta et al. [156] reported that the rate of change in wall shear stress encountered by collecting lymphatic vessels significantly affects the contractile response of lymphatic vessels to flow and that fluid shear stress has the capacity to regulate the coordination of lymphatic pumping activity between lymphangions. In vitro, even low, 0.5 or 1.0 dyn/cm^2^ shear stress has been shown to trigger the release of ATP from LECs, which results in an increase in eNOS via activation of the purinergic P2X/2Y receptor and enhanced Ca^2+^ signaling [157]. Thus, shear stress-dependent NO production by eNOS in LECs may support the coordination between shear stress and lymphatic pumping by controlling the contractility of SMCs, which is essential for removing excess interstitial fluid load and preventing oedemic conditions.

### Potential synergy between fluid flow-induced shear stress and other biological signals

Although the response to FSS itself in LECs can be sufficient to maintain the complex structure of the vessel necessary for lymphatic functions, LECs may also integrate biochemical and fluid shear stress to do so (Fig. 2A). There is evidence supporting that synergy between FSS and traditional biological factors such as VEGF-C promotes the remodeling of lymphatic vasculature [4]. For example, slow interstitial flow, with an average velocity of 4.2 m/s, synergizes in vitro with molecular factors promoting lymphatic vessel sprouting by enhancing the availability of matrix-bound growth factors to the cells [158]. Likewise, it was demonstrated more recently in a 3D in vitro model that interstitial flow with 1 m/s average velocity augments the effects of prolymphangiogenic molecular factors and determines the direction of lymphatic sprouting [159]. Intriguingly, FSS can also affect LECs by cooperating with S1P. S1P and one of its receptors, S1PR1, are commonly involved in individual and collective cell migration. As described above, the concentration of S1P is nearly zero in interstitial fluid, while it is high in blood and lymph, and LECs are the major source of lymph S1P. S1P can promote LEC sprouting in vitro in an S1PR1-dependent manner [160]. The study by Surya et al. [161] proposed a model in which S1P and FSS act synergistically during the development and remodeling of the lymphatic system. Previously, using an impinging flow chamber, the authors discovered that some LECs migrate upstream against flow direction [162]. Subsequently, they provided evidence that this upstream migratory phenotype requires both S1P and its receptor, suggesting that S1P may act as a biochemical cue in response to FSS. However, the mechanism by which FSS stimulates S1PR1 remains to be elucidated. Interestingly, another study reporting that LSS enhances VEGF-C signaling and LEC sprouting, further supporting LSS as a lymphangiogenic mediator, revealed that this process is directly antagonized by S1PR1 [163]. The authors proposed that S1PR1 may prevent the hyperactivation of LSS/VEGF-C signaling to halt the sprouting of lymphatic vessels. Moreover, they showed that S1PR1 controls the cytoskeletal and membrane localization of claudin-5 by inhibiting RhoA activity. Although it is not clear how LSS enhances VEGF-C signaling, it is plausible that LSS boosts the interaction between VEGFR3 and its coreceptor NRP2 or molecules such as VEGFR2 and integrin-α9. On the other hand, LSS may control the expression or localization of kinases or phosphatases modulating the phosphorylation of VEGFR3 or downstream signaling molecules.

Altogether, these findings indicate that synergistic effects between biomechanical and chemical cues control lymphatic biology and functions, and more studies will be needed to uncover novel synergies between FSS and unexplored biochemical factors, such as chemokines and cytokines.

### Mechanotransduction of fluid flow-induced shear stress in lymphatic vessels

Mechanotransduction is the process by which cells translate mechanical information into biological responses. The mechanotransduction of several shear stress responses in ECs, such as cell alignment, has been shown to require VEGFR2. However, the study of Bayens et al. [149] on differences in flow sensitivity between BEC and LEC revealed that VEGFR2 expression levels were comparable, unlike VEGFR3 expression, which was increased in LECs. FSS also stimulates activation of VEGFR3, as indicated by increased phosphorylation [149]. In vivo, augmentation of interstitial fluid volume mechanically stretches LECs, enhances VEGFR3 activation and induces LEC proliferation in a β1 integrin-dependent manner [164] (Fig. 2B) This is consistent with the role of some β1 integrins, such as α5β1, in mechanotransduction [165] and their capacity to associate with VEGFR3 and trigger its activation [166, 167]. Integrins have also been postulated to connect the extracellular environment with intracellular signaling by linking the extracellular matrix with the actin cytoskeleton inside LECs [165]. Furthermore, integrins have been implicated in lymph valve formation in vivo [168] and in lymphangiogenesis during pathological processes such as inflammation, tumor growth, and wound healing [169–172].

Several groups have focused their efforts on identifying flow-induced transcription factors mediating FSS responses in lymphatic vessels. An in vitro study showed that LECs become elongated and aligned under 4 dynes/cm^2^ LSS, while they were more cuboidal under 4 dynes/cm^2^ ¼ Hz OSS and that these alterations in LEC alignment and cytoskeleton reorganization were triggered by the transcription factors Forkhead box protein C2 (FOXC2) and PROX1 [147] (Fig. 2B). FOXC2 is essential for the maturation of lymphatic vessels and lymphatic valve development and PROX-1 functions as the master regulator of lymphatic development [107]. Specifically, OSS, but not LSS, upregulates FOXC2, whereas PROX1 is not affected. Loss of PROX1 abolishes the LEC response to OSS while enhancing cell elongation and alignment in response to LSS [147]. OSS coordinately induces Cx37 and calcineurin/NFAT activation in a PROX1- and FOXC2-dependent manner in LECs in vitro (Fig. 2B). Foxc2- deficient LECs exhibit disrupted cell‒cell junctions and a disorganized cytoskeleton, leading to an abnormal response to shear stress, cell hyperproliferation, and apoptosis. Moreover, this aberrant proliferation of Foxc2-deficient LECs in response to OSS is mediated by increased YAP/TAZ signaling [147, 173]. YAP and TAZ are transcription factors that were previously shown to regulate cell responses to mechanical cues such as stiff ECM, stretching and/or shear stress [174]. Thus, These findings suggest the essential role of FOXC2 in stabilizing collecting vessel quiescence through the coordination of cell‒cell junction maturation and responses to shear stress in regions of disturbed flow, such as valves. Another in vitro shear stress study confirms the effect of FSS on the upregulation of FOXC2, CX37, ITGA9, and GATA2 [175], which are known to be essential for valve-forming LECs in vivo [168, 176–178] (Fig. 2B). Notably, a recent study identified FOXP2 as a new flow-induced transcriptional regulator of collecting lymphatic vessel and valve development and a downstream effector of the flow-responsive FOXC2/NFATc1 pathway [179] (Fig. 2B).

An in vivo study identified FAT tumor suppressor homolog 4 (FAT4), a target gene of GATA2, as a key player in shear stress-dependent polarization of LECs [180] (Fig. 2B). Though FAT4 is clearly important for transducing laminar flow-induced signals in LECs, the mechanisms by which it transduces mechanical signals in endothelial cells remains to be elucidated. A study by Choi D et al. [181] investigated the molecular mechanisms underlying flow-induced lymphatic growth. They showed in vitro that LSS enhances the intracellular calcium level in LECs through the early signal and mediator of laminar flow, ORAI1 (calcium release activated calcium modulator 1), a pore subunit of the calcium release-activated calcium channel. ORAI1 activates key transcription regulators, including Klf-2 and Klf-4, which in turn upregulate VEGF-C, VEGF-A, and FGFR3, promoting LEC proliferation and survival, and concurrently downregulated the cell cycle inhibitor p57 (Fig. 2B). These in vitro findings were further validated in mice depleted of ORAI1, Klf-2 or Klf-4, which exhibited a reduction in lymphatic vessel density and development [181]. The same group further demonstrated that calcium-loaded calmodulin forms a protein complex with Klf2 (a master regulator of shear stress responses), and Prox1 binds to the promoters of Dtx1 and Dtx3L and activates their gene expression [182] (Fig. 2B). In turn, the Dtx1 and Dtx3L proteins form a Notch E3 ligase complex, which decreases Notch activity and promotes lymphatic sprouting [182] (Fig. 2B). Consistent with the role of calcium and ORAI1 in the early response to flow, a study showed that the magnitude of shear stress modulates the intracellular calcium dynamics in cultured LECs, and calcium mobilization is reduced through blockade of calcium release-activated calcium channels [183].

### Mechanosensing of fluid flow-induced shear stress in lymphatic vessels

As reviewed above, FSS is an essential regulator of the stabilization of mature lymphatics, adult lymphangiogenesis and biological functions of lymphatics. To date, two potential shear stress-sensing mechanisms have been identified in lymphatics. The complex involving mechanosensory receptors at the cell‒cell junctions on the LEC surface including PECAM, VE- cadherin, VEGFR2 and VEGFR3 has been proposed as a mechanosensing mechanism of shear stress in lymphatics (Fig. 2B). Such mechanosensing complexes have been well characterized in BECs [184, 185] and shown to trigger cell proliferation by activating the phosphatidylinositol-3- kinase (PI3K)/Akt pathway and cytoskeleton reorganization in response to FSS [186, 187] (Fig. 2B).

The involvement of PECAM-1 in lymphatic mechanosensing was revealed in mice with PECAM deficiency, which led to defective lymphatic remodeling, affecting processes such as valve formation [188]. Notably, VE-cadherin distribution within the cell membrane in BECs to has been shown be dependent on the spatial flow characteristics [189], but whether this is also observed in LECs remains to be demonstrated. VE-cadherin acts as an adaptor by binding to VEGFR2 and VEGFR3 [184], which are expressed on LECs and known to contribute to lymphatic development and adult lymphangiogenesis [107]. Consistent with the role of VEGFR3 as a flow sensor, VEGFR3 expression in LECs changes in response to flow characteristics [115], and as discussed above, it is a key determinant of flow sensitivity in ECs [149]. Thus, considering the individual contributions of PECAM, VE-cadherin, VEGFR2 and VEGFR3 to lymphatic remodeling, stabilization, and flow sensitivity, it is possible that together they form a mechanosensing complex in LECs to respond to FSS. However, further investigations are required to validate this mechanosensing mechanism.

The molecular sensor that senses flow-induced mechanical force in LECs and activates ORAI1-mediated calcium entry has remained unknown until the recent follow-up study by Choi et al. [190] which identified Piezo-1 (Piezo type mechanosensitive ion channel component 1) as an upstream mechanosensor of ORAI1 that triggers the mechanotransduction signal controlling lymphatic expansion in response to external physical stimuli (Fig. 2B). Piezo1 is sensitive to alterations in membrane tension [191] and has been reported to be essential for sensing fluid shear stress by BECs [192]. Similarly, Piezo-1 has been reported to be mechanically activated in LECs and lymphatic vessels and to serve as a mechanical force sensor controlling lymphatic valve development [193] and maintenance in adult lymphatics [194]. In a later study by Choi et al. [194] depletion of Piezo1 in cultured LECs significantly reduced the OSS-induced upregulation of lymphatic valve signature genes, including GATA-2 and Foxc2, while its overexpression in LECs or activation using the chemical agonist Yoda-1 upregulated the lymphatic valve genes in the absence of OSS. In their more recent study, Choi et al. [190] reported that Piezo1 overexpression alone recapitulates the laminar flow-induced upregulation of OraI1 downstream genes, such as Dtx1, Dtx3L, and Klf2, and downregulation of Notch. OraI1 stimulation alone was sufficient for LEC mechanotransduction in the absence of laminar fluid flow or Piezo1 activation. These findings demonstrate that Piezo-1 is an upstream activator of OraI1 in the laminar flow-activated mechanotransduction pathway in LECs. The authors also showed that Piezo-1 is essential for the maintenance of lymphatic vessels in adult mice, as evidenced by a reduction in mesenteric and dermal lymphatics after specific deletion of Piezo-1 in lymphatics postnatally [190]. Moreover, Piezo-1 exhibited prolymphangiogenic properties in animal models when activated by the agonist Yoda-1, and these properties translated into therapeutic benefits in an animal model of surgically induced lymphedema [190]. The discovery of the essential role of Piezo-1 in lymphatic development and maintenance also provides some mechanistic explanation for the generalized lymphatic dysplasia and dysfunction observed in patients with mutations in Piezo-1 [195, 196].

### Concluding remarks

Considering the vital functions that the lymphatic vessels play and the increasing list of their contributions to human diseases, it is imperative to improve our understanding of how these vessels sense and respond to environmental changes encompassing not only biochemical but also mechanical signals. As described in this review, recent research on biomechanical signals further supports the concept that lymphatic vessels are not “inert tubes” but rather highly plastic vasculature structures capable of responding to biomechanical changes and adapting their properties, such as permeability and contractibility, to changes in their surrounding microenvironment. However, many questions remain unresolved, including how biomechanical forces contribute to controlling lymphangiogenesis in pathological conditions such as cancer and metabolic and inflammatory diseases. However, identifying the specific role of biomechanical signals distinct from that of biochemical signals might be challenging because numerous downstream molecules are shared by both signals. It would also be interesting to investigate whether alterations in mechanosensing and/or mechanotransduction in lymphatics are associated with diseases that exhibit altered fluid flow. To this end, new tools may have to be developed to allow the visualization and accurate measurement of lymph flow. Although evidence supports the control of lymphatic functions by FSS, it is likely that lymphatic vessels are exposed to more than one biomechanical cue at a given time, particularly in a pathological setting, where the matrix composition or stiffness is expected to change. Thus, we need to understand how lymphatic vessels integrate multiple biomechanical signals and respond to them to avoid potential deleterious effects.

More research is needed to further understand mechanotransduction and mechanosensing in lymphatic vessel during homeostasis and pathological conditions, and the results of such research may lead to the discovery of new promising therapeutic approaches. As an example, targeting the mechanical force sensor, Piezo-1 has shown encouraging preclinical results for the treatment of secondary or primary lymphedema by promoting the growth of functional lymphatic vessels.
